# CT and 3 Tesla MRI in the TN Staging of Colon Cancer: A Prospective, Blind Study

**DOI:** 10.3390/curroncol29020091

**Published:** 2022-02-13

**Authors:** Søren R. Rafaelsen, Claus Dam, Chris Vagn-Hansen, Jakob Møller, Hans B. Rahr, Mikkel Sjöström, Jan Lindebjerg, Torben Frøstrup Hansen, Malene Roland Vils Pedersen

**Affiliations:** 1 Department of Radiology, Vejle Hospital, University Hospital of Southern Denmark, 7100 Vejle, Denmark; claus.dam@rsyd.dk (C.D.); chris.aksel.vagn-hansen@rsyd.dk (C.V.-H.); Jakob.Moeller@rsyd.dk (J.M.); Malene.Roland.Vils.Pedersen@rsyd.dk (M.R.V.P.); 2 Danish Colorectal Cancer Center South, Vejle Hospital, University Hospital of Southern Denmark, 7100 Vejle, Denmark; Hans.Rahr@rsyd.dk (H.B.R.); jan.lindebjerg@rsyd.dk (J.L.); Torben.Hansen@rsyd.dk (T.F.H.); 3 Department of Regional Health Research, University of Southern Denmark, 5000 Odense, Denmark; 4 Department of Surgery, Vejle Hospital, University Hospital of Southern Denmark, 7100 Vejle, Denmark; mikkel.Sjostrom@rsyd.dk; 5 Department of Pathology, Vejle Hospital, University Hospital of Southern Denmark, 7100 Vejle, Denmark; 6 Department of Oncology, Vejle Hospital, University Hospital of Southern Denmark, 7100 Vejle, Denmark

**Keywords:** colon cancer, t-staging, lymph node, CT, MRI, DWI

## Abstract

(1) Background: Computer tomography (CT) scanning is currently the standard method for staging of colon cancer; however, the CT based preoperative local staging is far from optimal. The purpose of this study was to investigate the sensitivity and specificity of magnetic resonance imaging (MRI) compared to CT in the T- and N-staging of colon cancer. (2) Methods: Patients underwent a standard contrast-enhanced CT examination. For the abdominal MRI scan, a 3 Tesla unit was used, including diffusion weighted imaging (DWI). Experienced radiologists reported the CT and MRI scans blinded to each other and the endpoint of the pathological report. (3) Results: From 2018 to 2021, 134 patients received CT and MRI scans. CT identified 118 of the 134 tumors, whereas MRI identified all tumors. For discriminating between stage T3ab and T3cd, the sensitivity of CT was 51.1% and of MRI 80.0% (*p* = 0.02). CT and MRI showed a sensitivity of 21.4% and 46.4% in detecting pT4 tumors and a specificity of 79.0% and 85.0%, respectively. (4) Conclusion: Compared to CT, the sensitivity of MRI was statistically significantly higher in staging advanced T3cd and T4 tumors. MRI has the potential to be used in the treatment planning of colon cancer.

## 1. Introduction

Colon cancer is among the most prevalent cancers [1]. In the past, surgery was the only treatment; around half of the patients recurred with incurable disease. With adjuvant chemotherapy, a moderate improvement in survival can be achieved for some patients with stage II and III colon cancer [2]. In rectal cancer, neoadjuvant treatment has been shown to be effective [3,4]. Neoadjuvant chemotherapy holds the potential to improve the outcome of advanced colon cancer with effective control and reduction of tumor size [5,6,7].

Not all patients benefit from chemotherapy before surgery, because the preoperative staging of colon cancer is not optimal with CT [8]. Patients whose tumor is underestimated before surgery are at risk of needing subsequent chemotherapy, which may be less effective than up-front chemotherapy. In addition, some patients may be eligible for radical surgery but, if the CT scan has underestimated the spread of the tumour, do not undergo this treatment. The goal of the MRI scan is to provide a more accurate preoperative diagnosis and, hence, to optimize treatment with potentially increased survival.

CT scanning is currently the national standard method for determining disease stage during treatment planning [8], but the CT based preoperative staging could be improved [8]. Advanced colon tumors are defined by >5 mm growth beyond the bowel wall [5]. The prevalence of advanced tumors was just above 40% in a CT study from 2013 [9] and 34% in a recent MRI study [10]. The sensitivity and specificity in the staging of advanced colon tumors in the CT study was approx. 70% and 80%; in the small MRI pilot study, the values were 89% and 96%, respectively.

MRI of the rectum has been documented to be more accurate than CT scanning [11]; it possesses the advantage of tumor diffusion measurement, which gives an estimate of cell density. There has been some reluctance to introduce MRI of patients with colon cancer, since the colon contains more peristalsis and movement can result in artefacts. However, in recent years MRI has become faster and, thus, less sensitive to movement. MRI has been proposed by others as a new method in selecting patients who could benefit from neoadjuvant treatment [12,13,14,15]. However, there are only a few MRI studies on diagnostic accuracy. Some evaluate MRI without comparing to CT [16,17], and most of the comparing studies are retrospective [12,18,19]. A limited number of comparative prospective studies are available, and only with few patients [15,20]. Furthermore, the literature is scarce on adding diffusion-weighted MRI in the preoperative local staging of colon cancer. The advent of 3 Tesla MRI presents the opportunity to scan in a more time-efficient manner. Prior to a multicenter 3 Tesla whole body study, we sought to evaluate the possible advance of the method in the local staging of colon cancer as it has been done with rectal cancer.

The main purpose of the study was to investigate the applicability of 3 Tesla MRI in colon cancer by assessing the sensitivity and specificity, to clarify whether it is superior to CT scanning for the T-, N-staging. A secondary aim was to elucidate the additional results of diffusion weighted MRI.

## 2. Materials and Methods

### 2.1. Study Design

This study is a prospective, observational, blind study. A colon cancer patient treated with radical surgery at our hospital was involved in the evaluation of the study protocol prior to approval and patient enrollment. All participants received a standard CT scan performed using a Philips Brilliance 64-slice CT scanner (Philips, Eindhoven, The Netherlands) with dynamic dose modulation. A breath-hold technique was applied using the following parameters: 120 kV; 93 mA; rotation time = 0.5 s; pitch = 1.17; collimation = 0.6 mm; increment = 2. The effective dose was 8–9 mSv. The CT was performed in portal phase with IV contrast 100 mL iomeprol (Iomeron, Bracco, Milan, Italy) 300 mg I/mL using an automatic injector OptiVantag DH (Mallinckrodt, Cincinnati, OH, USA) with an injection rate of 4 mL/sec. The axial images from 3 mm CT slices underwent review by an experienced gastrointestinal radiologist (>10 years of experience). The radiologists also evaluated the obtained coronal and sagittal reformats CT images. The tumor location was noted in the request form. The CT radiologist was blinded to the MRI result.

For the MRI scan, the equipment was a big bore Ingenia 3 Tesla MRI unit (Philips Medical Systems, Best, The Netherlands) with an aperture diameter of 70 cm. Anterior and posterior body coils were used. Initially, a coronal T2w image of the abdomen was obtained from the diaphragm to the lower pelvis; then, a coronal T2w sequence to localize the tumor and plan the high-resolution images. In detail: Coronal T2: Voxel size: 1.2 × 1.2 × −5 mm, FOV: 440 × 440 × 221 mm, matrix: 368 × 368, slice gap: 1 mm, TE/TR: 132/2725, scan time: 2.30 min. Sagittal T2: Voxel size: 1.2 × 1.2 × −6 mm, FOV: 440 × 440 × 221 mm, matrix: 368 × 368, slice gap: 1 mm, TE/TR: 132/2725, scan time: 1.50 min. DWI b0-b800: Voxel size: 3 × 3 × 5 mm, FOV: 450 × 359 × 179, matrix: 152 × 117, slice gap: 1 mm, TE/TR: 80/1658, scan time: 2.30 min. Axial T2: Voxel size: 0.8 × 0.8 × 3 mm, FOV: 325 × 325 × 104 mm, matrix: 408 × 408, slice gap: 0.5 mm, TE/TR: 120/4564, scan time: 2.53 min. DWI b0-b1000 zoom: Voxel size: 2.6 × 2.68 × 4 mm, FOV: 190 × 116 × 104, matrix: 72 × 48, slice gap: 0, TE/TR: 86/4640, scan time: 2.50 min. The last T2w sequences axial to the tumor were planned together with a gastrointestinal radiologist. MRI scan of the tumor process was performed with thin slices. The DWI sequences with b0 and b1000 were also obtained on the angulated axial images. ADC maps of the isotropic images were created automatically by the Philips software. The patients were scanned in the supine position, headfirst with an average scan time of 25 min. No intravenous, oral or rectal contrast medium was administered. The experienced gastrointestinal MRI radiologist was blinded to the CT results. To ensure the blinding, the images were sent directly to the IntelliSpace portal file and the MRI scan was released to the department’s Picture Archive Communication System (PACS) when the CT report was finalized.

Radiologists with more than 10 years’ experience reported the CT and MRI scans blinded to each other and the pathological description. Prior to the present study, both the CT and MRI radiologist had received feed-back from the pathologist at weekly colorectal multidisciplinary team (MDT) meetings for five years [21]. All images were evaluated using an Easyviz Impax PACS workstation (Medical In-sight, Valby, Denmark) with a 21.3” monitor CCL358i2 from TOTUKU, JVCENWOOD Cooperation, Kanagawa, Japan. An ADC map in grayscale was automatically generated by the Philips system using a mono-exponential decay model. The observers recorded the tumor size, T-stage, extent of extramural tumor invasion (ETI), presence of metastatic lymph nodes, presence of extramural vascular invasion (EMVI), and ADC value of the colonic tumor and the largest lymph node. Size was reported as the largest tumor diameter in one of the perpendicular planes. T-stage assessed by MRI was evaluated according to the TNM classification. ETI was defined as maximal tumor outgrowth from the intestinal wall in millimeters. T4 tumors and T3 tumors with >5 mm ETI (T3cd–T4) were classified as locally advanced and T1, T2, and T3 tumors with an ETI of ≤5 mm were classified as early (i.e., non-locally advanced). Regional lymph nodes, regardless of metastatic status, were identified using both T2W and DWI sequences. After detection, lymph nodes with 10 mm size (short axis) alone or with >5 mm size (short axis) combined with an irregular border and/or inhomogeneous signal were defined as metastatic. EMVI was considered present if the tumor invaded a pericolic vessel. ADC values were reported as the mean ADC acquired by manual drawing of a precise region of interest (ROI) on the ADC map delineating the most restrictive part of the solid tumor on a single slice, avoiding intraluminal air.

The CT and MRI reports were presented at the MDT conference where the blinding was lifted, and treatment was scheduled. Surgery was performed within a median of one week after the MDT. The resected specimens were transferred to the Department of Pathology and fixed for two days in neutral, buffered formalin. After fixation, all tumors were sliced transversally. The tumor size and deepest penetration from the outer edge of tunica muscularis were measured on the slices and confirmed microscopically. The resected specimens were classified according to the pTNM system by an experienced gastrointestinal pathologist (JL). The peritoneal surface was considered involved (pT4a) if viable cancer was noticed outside the peritoneal lining or infiltrating adjacent organs (pT4b). The pathologist was blinded to the CT and MRI findings. The endpoint of the study was the histopathological staging of the surgical specimen.

### 2.2. Inclusion of Trial Participants

Patients >18 years of age diagnosed with colon cancer, typically by colonoscopy, received oral and written information about the study at the endoscopic section of the Department of Surgery, University Hospital of Southern Denmark, Vejle. The first contact occurred after the colonoscopy. At the Department of Radiology, the information was repeated and any questions were answered before the patient provided written consent.

The exclusion criteria were use of a pacemaker, implanted drug pumps or nerve stimulators, severe claustrophobia, prior radiotherapy/chemotherapy, or any other condition or disease as assessed by the investigator made it inappropriate for the patient to participate in the trial.

Written informed consent was obtained from all subjects involved in the study. The study was conducted according to the guidelines of the Declaration of Helsinki, and the Regional Committees on Health Research Ethics for Southern Denmark approved the study.

### 2.3. Data

The patient’s medical record was the source of data for inclusion and exclusion criteria, demographic data and the course of treatment. Only data needed to achieve the results of the study were recorded, namely gender, age and tumor stage based on radiology and pathology reports. Information on the subjects was protected under the General Data Protection Regulation and the Health Act. The study was approved by the Regional Committees on Health Research Ethics for Southern Denmark (protocol code: S-20180078 and date of approval: 31 August 2018); the data processing was approved by the Region of Southern Denmark. A database was generated in REDCap administered by the Open Patient data Explorative Network. The analysis was performed using STATA v 16.1 (TX, USA). The study was registered with ClinicalTrials.gov, Identifier: NCT04391933.

### 2.4. Statistical Analysis

According to the null hypothesis (H0), there was no difference in sensitivity and specificity between CT and MRI scans in the detection of advanced colon cancer. The alternative hypothesis (H1) was that the MRI scan showed a significantly better sensitivity of 10–20%. Since the introduction of colorectal cancer screening has reduced the rate of advanced tumors, we could not expect a prevalence of 43%, as in the previous study. The prevalence of advanced tumors was, therefore, set at approx. 30%. With a power of 80%, a sample size of 100 patients was needed to detect a significant difference with *p* < 0.05.

The sensitivity and specificity of the CT and MRI scans with 95% confidence intervals (CI) were calculated using the histopathology report as the endpoint. Descriptive statistics were applied. McNemar’s test was used to compare the sensitivity and specificity in the evaluation of the two diagnostic modalities [22]. A *p* value < 0.05 was considered statistically significant. Comparison of proportions was performed using the X2 test or, when appropriate, Fisher’s exact test.

## 3. Results

### 3.1. Patient Characteristics

**Figure 1:**
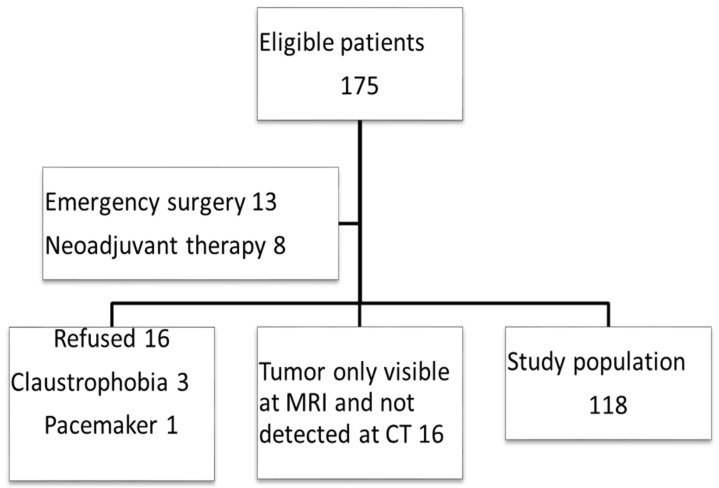
STARD Flow chart of patient inclusion.

From 1 September 2018 to 31 December 2020, a total of 175 patients were eligible for the study. As it appears in the flow chart, 57 patients were excluded Figure 1.

**Figure 2:**
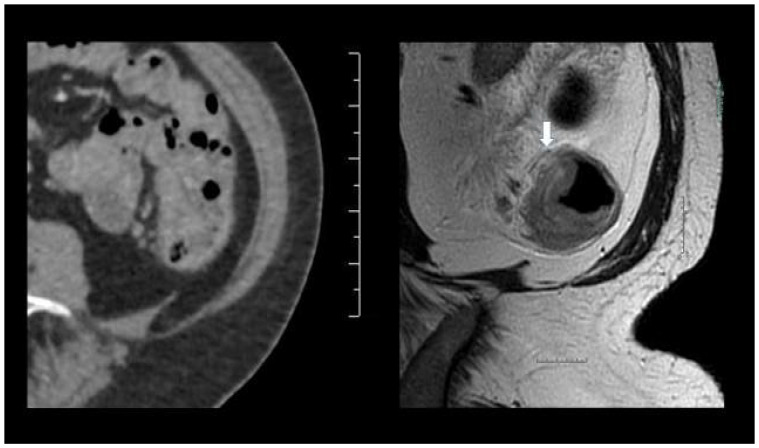
(**left**) The CT scan did not identify the colonic tumor, although the request indicated a tumor in the descending colon detected at colonoscopy. (**right**) The MRI scan showed a semicircumscript tumor at the mesenteric side of the descending colon (cT3a, N1,V0), confirmed at the histopathological specimen.

CT failed to detect 16 of the tumors (pT3, N = 8; containing lymph node metastases, N = 6; EVI, N = 2), whereas MRI identified all the included tumors, Figure 2.

This provided a comparable study population of 118 patients (Table 1).

Nine patients harbored synchronous liver metastases and four showed lung metastases. Immunohistochemistry of tumors with deficient mismatch repair (MMR) protein revealed PMLH1 (protein MutL homolog), N = 15, PMSH 2(protein MutS homolog), N = 7, PMSH6, N = 5, PMS2 (protein MutS), N = 9. The remaining were proficient MMR tumors.

### 3.2. Sensitivity and Specificity

The sensitivity of correct identifying advanced T3c-T4 tumors was 29% higher for MRI (McNemar’s test). The CT and MRI had comparable sensitivity and specificity in diagnosing lymph node involvement. For the evaluation of extravascular involvement, CT possessed a sensitivity of 35.0%, whereas MRI tended to show a higher sensitivity of 50.0% (Table 2).

**Figure 3:**
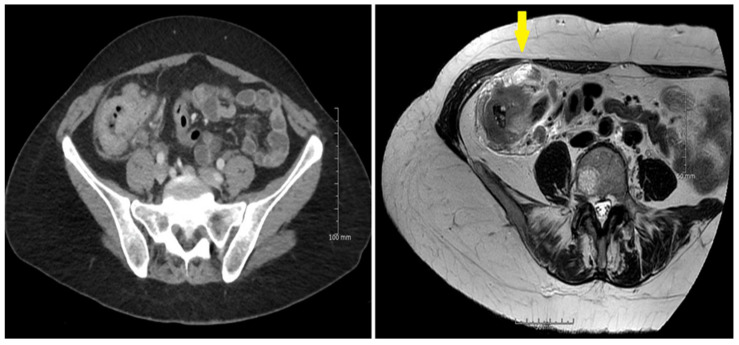
(**left**) The serosal involvement of a large cecal pT4a,N1,V2 tumor was not identified by CT. (**right**) MRI identified the serosal involvement seen at the anterior part of the tumor (arrow). MRI also spotted lymph node metastases as well as extra mural vascular involvement, confirmed at the histopathological examination.

**Figure 4:**
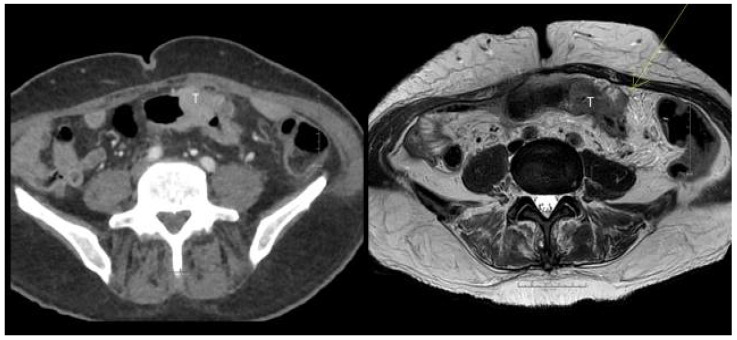
(**left**) CT image of a tumor (T) in an elongated sigmoid sling. Reported as a large T2,N0,V0 tumor. (**right**) MRI reported a T3c,N1,V2 tumor with an outgrowth from the colonic wall of 7 mm (arrow). Histopathology confirmed all the MRI findings.

MRI identified more T4 tumors compared to CT, however, with an overlap of the confidence limits. Figure 3 and Figure 4.

### 3.3. Diffusion Weighted MRI

The mean primary tumor ADC tended to be lower in patients with distant metastases than in those without (0.645 vs. 0.791, *p* = 0.13). The diffusion restriction was significantly lower in mucin-containing tumors, with an ADC of 1.08 vs. 0.767 (*p* < 0.001). The same was seen for PMS2, with an ADC of 0.914 vs. 0.777 (*p* < 0.035).

## 4. Discussion

CT failed to identify 16/134 (12%) colon tumors, half of which were pT3 tumors. A similar finding has been reported previously in a retrospective study of 127 patients with colon cancer unidentified in 20% of abdominal CT examinations in patients subsequently diagnosed with colon cancer by colonoscopy. They found absence of fat stranding, vascular engorgement or lymphadenopathy, and an average tumor length of 3.3 cm to be contributing factors for failure of detection [23]. The use of DWI helped as an eye catcher and could be an explanation of the superiority of MRI in this context. The clinical importance of staging all tumors cannot be taken lightly, since half of the CT unidentified tumors breached the bowel wall.

The main new finding of the present comparative prospective study was that MRI showed a 29% significantly higher sensitivity compared to CT in discriminating between pT1-T3b and pT3c-T4 tumors. The specificity was 91% and 80.0% for MRI and CT, respectively, but the difference did not reach statistical significance. There was a small overlap of the two CI intervals. A study of 29 patients reported an MRI sensitivity of 77–92% and that of CT at 69% in relation to pT3c-T4 tumors [12]. Hunter et al. found a non-significant higher sensitivity of MRI compared to CT (75% vs. 70%) for reader 1 and, conversely, lower sensitivity for reader 2. A retrospective study including 116 patients with sigmoid and descending colon cancer compared the staging of MRI and CT using McNemar´s test and found that MRI may offer superior diagnostic performance over CT in detecting advanced colonic disease [18]. Song et al. using a 1.5 Tesla MRI unit found a sensitivity of 45–62% in diagnosing T3c-T4 tumors [17]. Given the relatively poor sensitivity of 1.5 Tesla studies in detecting of T3c-T4 tumors, 3 Tesla is superior with a higher sensitivity of 80% in the present study and up to 86% by Park et al. in 38 patients [19]. An inter-observer study of 29 patients found a higher kappa value for MRI than CT (0.82 vs. 0.39) in categorizing T3c/T4 colon tumors [19].

We found the ability of CT and MRI to diagnose bowel wall penetration to be almost identical. Our finding of a sensitivity of MRI of 78% in detecting T3 tumors is high com-pared to the finding of 42–74% by Hunter et al. [13]. On the other hand, our specificity was lower, with 61% compared to the MRI specificity of a two-observer study of 75–83% [13]. Nerad et al. found a sensitivity ranging between 72% and 91% [16]. Another retrospective study of 116 patients found a higher specificity of MRI compared to CT regarding bowel wall penetration. The study included only tumors from the descending and sigmoid colon [18]. It can be difficult to detect a small outgrowth of a few millimeters. Song et al. also found a high sensitivity of MRI in detecting T3/4 tumors [17]. Although we did not perform an inter-observer study, a previous inter-observer study found a kappa value of 0.79 for the T-staging of colon cancer using MRI and a kappa of 0.64 by CT [12].

Our 28 T4 tumors were identified twice as often using MRI compared to CT (Table 2), but with a big overlap of the 95% CI. Our results were inferior to a previous study with a sensitivity in detecting T4 tumors of 83%, although with a smaller number of colonic tumors (N = 32) [24].

The nodal staging was in accordance with previous publications [12,13,16,17]. In our study, the lymph nodes with a size of (short axis) 10 mm alone or with a size of (short axis) >5 mm combined with an irregular border and/or inhomogeneous signal were defined as metastatic. The definition of lymph node metastasis we used has shown promising results to predict lymph node status in colon cancer [25]. In nodal staging, a relatively high sensitivity is often related to a low specificity and vise-versa. Artificial intelligence (AI) has recently shown promising results in the nodal staging of rectal cancer [26]. However, no AI studies are available on lymph nodes metastases from colonic cancer.

In the present study, MRI diagnosed EMVI with a higher sensitivity than CT, although detecting only half of the cases. Liu et al. reported a sensitivity of 64% using MRI compared to 40% by CT [18]. Hunter et al. also used a 3 Tesla scanner and found a similar higher sensitivity using MRI [13].

Tumor ADC tended to be lower in patients with distant metastases, the finding of which needs to be confirmed in larger long-term follow-up studies. Low ADC-values within the primary tumor possess the potential to predict lymph node metastases or distant metastases [27]. Histogram analysis of ADC MRI also seems to have potential as a prognostic tumor marker of colorectal cancer [28]. Tumors containing mucin showed less diffusion restriction, which is in accordance with a study on 62 rectal cancer patients [29]. The clinical importance is not clear. A new study found rectal mucinous adenocarcinoma to respond poorly to neoadjuvant treatment [30]. Tumors with PMS2 showed less diffusion restriction on MRI, which supports the finding of [31] showing higher mucinous content in PMS2 colonic tumors. Some authors suggest a more aggressive behavior of these tumors [32], and others do not [33]. Patients with deficient MMR may hold the potential to respond to neoadjuvant immunotherapy, but this needs to be validated in larger studies [34].

It is a limitation of our study that a number of patients were excluded from the study population and that inter-observer variation was not evaluated. We did not use intravenous contrast or spasmolytic agents, which is not necessarily a disadvantage. Park et al. did not improve their accuracy using gadolinium-enhanced sequences for the diagnosis of T3c-T4 tumors [19]. We did not analyze the impact of the MRI finding on the MDT decision. The radiologist assisted angulation in the perpendicular T2w sequences is a limitation, but the image quality was improved and comparable to that of the angulation in rectal MRI [11]. If the angulation was very steep, a decreased image quality was seen, and we did not quantify this aspect. The examination time was up to or less than 25 min. This was intentionally constrained to reduce patient discomfort.

We hope that future patients will benefit from the higher sensitivity of MRI scanning and, thus, avoid radiation from the standard CT scan used today. This, however, will require further validation. The multicentric Streamline C study using 1.5 Tesla whole-body (WB) MRI found an accuracy similar to that of standard CT in both local and distant staging in the non-inferiority setting; the authors found WB MRI reduced the number of required extra scans, as well as the total assessment time and cost [35]. MRI makes more sense, if it is used as a one-stop-shop, including liver imaging, as local staging without staging of liver and lung staging could not serve as the basis of a treatment decision. A national multicenter study comparing 3 Tesla WB MRI with CT is now ongoing, including a non-inferiority evaluation of the M-stage, although DWI with 3 Tesla MRI can be challenging. Although the MRI examination time for local staging was relatively short, the patient inconvenience of MRI, as well as the possible clinical benefit of using WB MRI in the follow-up, also needs to be addressed in future studies.

## 5. Conclusions

MRI identified colonic tumors more often than CT. Compared to CT, the sensitivity of MRI was higher in the staging of advanced T3cd and T4 tumors. In the treatment planning of colon cancer, MRI could be advantageous.

## Figures and Tables

**Table 1:** Patient characteristics.

	N = 118 (%)
Gender	
Female	56 (47.5)
Male	62 (52.5)
Age	70.6 years [range 39–91]
Mean tumor length	4.5 cm [range 1.2–11.0]
Location of colon tumor	
Cecum	18 (15.3)
Ascending	36 (30.5)
Transverse	14 (11.9)
Descending	10 (8.5)
Sigmoid	40 (33.8)
Pathology	
pT1	3 (2.5)
pT2	25 (21.1)
pT3	62 (52.5)
pT4	28 (23.7)
pN1-2	55 (46.7)
pV2	40 (33.9)

**Table 2:** Study results, sensitivity and specificity of CT and MRI.

Tumor Stage	CT Sensitivity % (95% CI)	CT Specificity % (95% CI)	MRI Sensitivity % (95% CI)	MRI Specificity % (95% CI)	*p*
T1-2 vs. T3-4	61.8 (50.8–71.7)	85.7 (66.4–95.5)	77.8 (67.5–85.6)	60.9 (38.9–79.5)	ns
T1-3b vs. ≥T3c	51.1 (36.0–66.1)	80.8 (69.6–88.8)	80.0 (65.0–89.9)	91.8 (82.4–96.6)	*p* = 0.02 *
T1-3d vs. T4	21.4 (9.0–41.5)	94.3 (86.8–97.9)	46.4 (28.0–65.8)	94.4 (86.9–97.9)	ns
Nodal stage ±	65.5 (51.3–77.4)	50.0 (37.2–62.8)	58.2 (44.1–71.1)	50.0 (37.2–62.8)	ns
EMVI ±	35.0 (21.1–51.7)	82.0 (71.4–89.5)	50.0 (34.1–65.9)	81.8 (71.0–89.4)	ns

* Difference in sensitivity between CT and MRI.

## Data Availability

The data presented in this study are available on request from the corresponding author.
